# Anti-Neuroinflammatory Effect of Ombuin from *Rhamnus erythroxylon* Pall. Leaves in LPS-Induced BV-2 Microglia by Targeting Src and Suppressing the PI3K-AKT/NF-κB Signaling Pathway

**DOI:** 10.3390/ijms25168789

**Published:** 2024-08-13

**Authors:** Yanjie Bian, Nan Qiao, Suyun Han, Jixiang Gao, Xiaofang Lv, Lihuan Yuan, Linjing Zhang, Zuofu Wei

**Affiliations:** School of Life Science, Shanxi Normal University, Taiyuan 030006, China

**Keywords:** ombuin, BV-2, neuroinflammation, LPS, Src

## Abstract

The leaves of *Rhamnus erythroxylon* Pall. are widely used as tea substitutes in northwest China for their fragrant aroma, anti-irritability, and digestion-enhancing properties. Ombuin, a main flavonoid compound found in the leaves, exhibited notable anti-inflammatory and antioxidant effects. However, its potential role in treating neuroinflammatory-related diseases remains unexplored. Thus, this study aims to evaluate the anti-neuroinflammatory effects of ombuin and to explore the underlying molecular mechanisms. According to our findings, ombuin dramatically reduced the release of interleukin-6 (IL-6), tumor necrosis factor-α (TNF-α), IL-1β, nitric oxide (NO), and reactive oxygen species (ROS) in lipopolysaccharide (LPS)-stimulated BV-2 microglia. Further analysis, including transcriptomics, network pharmacology, molecular docking, and cellular heat transfer assays, revealed that Src was a direct target of ombuin. Western blot analysis showed that ombuin effectively suppressed Src phosphorylation and inhibited the downstream expressions of p-PI3K p85, p-AKT1, p-IKKα/β, p-IκBα, and nuclear factor κB (NF-κB). Meanwhile, the repression of Src significantly reversed the anti-neuroinflammatory activity of ombuin. Our results identified Src as a direct target of ombuin and implied that ombuin exerted an anti-neuroinflammatory effect by inhibiting Src phosphorylation and suppressing the activation of the PI3K-AKT and NF-κB pathways, which might provide an alternative therapeutic strategy for neurodegenerative diseases.

## 1. Introduction

Neuroinflammation occurs in all immune activities within the central nervous system and is associated with the pathogenesis of Alzheimer’s disease (AD) and other neurodegenerative diseases [1,2,3]. The initiation of neuroinflammatory responses is associated with microglia, which are the innate immune cells of the central nervous system [4]. Under normal conditions, microglia undergo tissue repair, remove cell debris, and maintain homeostasis [5]. However, inflammatory stimulation overactivates microglia, inducing the massive release of pro-inflammatory cytokines such as interleukin-1β (IL-1β), interleukin-6 (IL-6), and tumor necrosis factor-α (TNF-α), as well as neurotoxic substances like reactive oxygen species (ROS) and nitric oxide (NO). These factors can seriously accelerate neuronal damage, leading to neuroinflammation and neurodegenerative diseases [6,7,8].

Lipopolysaccharide (LPS), a component of the outer membrane of Gram-negative bacteria, is commonly used to activate microglia by binding to Toll-like receptor 4 (TLR4), thereby activating various inflammatory signaling pathways, such as nuclear factor κB (NF-κB) and mitogen-activated protein kinase (MAPK) [9,10]. Additionally, the phosphatidylinositol 3-kinase (PI3K)/Akt pathway is also involved in LPS-induced NF-κB activation in BV-2 cells [11]. Due to its control over the expression of numerous pro-inflammatory genes and its ability to be activated by various upstream signals, NF-κB is considered a central hub in the inflammatory process [12]. NF-κB is often targeted for the treatment of a variety of diseases, as it can be blocked by natural or artificial phytochemicals and immunosuppressive drugs [13]. Given the crucial role of microglial activation in neuronal injury and neuroinflammation, numerous studies have endeavored to identify its potential targets [14]. Among these targets, Src, a tyrosine kinase receptor closely associated with neuronal damage and inflammatory responses and involved in the occurrence and development of degenerative diseases, has garnered significant attention [15,16]. Src is expressed in the brain and exhibits diverse physiological functions, such as mediating synaptic plasticity, facilitating neuronal modulation, regulating cell proliferation, and controlling cellular signal transduction related to acute inflammatory responses [17,18]. It has been reported that Src activation regulates nicotinamide adenine dinucleotide phosphate (NADPH) oxidase activation, which subsequently stimulates ROS production and activates the downstream PI3K-Akt signaling pathway, leading to the activation of pro-inflammatory factor gene expression [19]. Consequently, the inhibition of microglial activation and the suppression of pro-inflammatory mediators and cytokine production are considered promising strategies for alleviating neurological disorders.

Natural bioactive compounds possess antioxidant, anti-inflammatory, and non-toxic properties, which have been reported to be effective in preventing neuroinflammatory disorders and exhibit immense potential in the treatment of neurodegenerative diseases [20,21,22,23]. The leaves of *R. erythroxylon* Pall., widely used as tea substitutes in China, are popular due to their fragrant aroma and beneficial effects such as eliminating irritability, aiding digestion, and clearing heat. Ombuin, a major flavonoid found in these leaves, has been reported to exhibit antibacterial, anti-inflammatory, and antioxidant activities in vitro [24,25,26]. However, the effect of ombuin on microglia-mediated neuroinflammation has not been previously reported. Thus, we evaluated the anti-neuroinflammatory ability of ombuin in LPS-stimulated BV-2 cells and explored the underlying molecular mechanism, which may offer an alternative therapeutic strategy for neuroinflammation.

## 2. Results

### 2.1. Ombuin Inhibited LPS-Induced Neuroinflammation in BV-2 Cells

**Figure 1:**
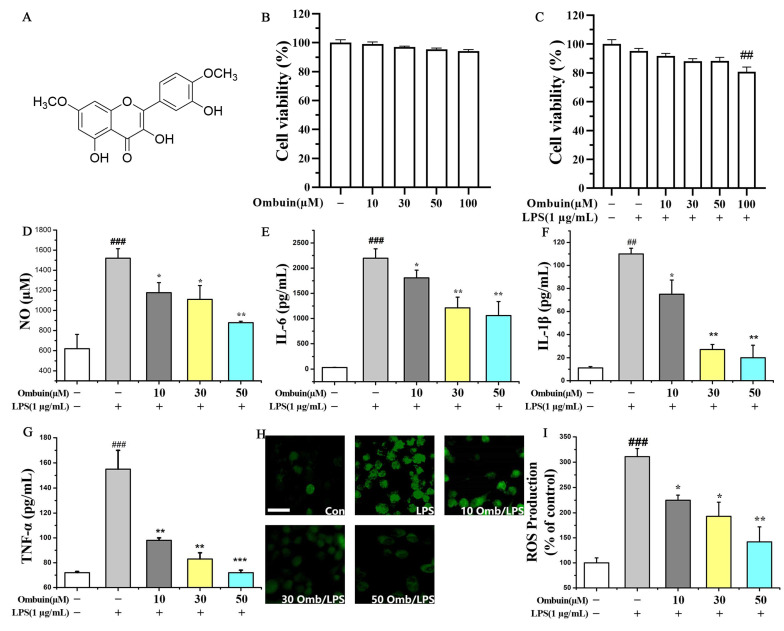
Ombuin inhibited LPS-induced neuroinflammation in BV-2 cells. (**A**) Chemical structure of ombuin. (**B**,**C**) Effects of ombuin on cell viability in the presence or absence of LPS. (**D**–**I**) The production of NO (**D**), IL-6 (**E**), IL-1β (**F**), TNF-α (**G**), and ROS (**H**,**I**) in LPS-induced BV-2 cells. After pretreatment with a series of ombuin concentrations for 1 h, BV-2 cells were stimulated with 1 μg/mL LPS for 24 h and then assayed by MTT, Griess, ELISA, and ROS detection assays. The scale bar represents 20 μm in (**H**). All values are expressed as the mean ± SEM for three independent experiments. ## *p* < 0.01 and ### *p* < 0.001 indicate significance compared with the control group; * *p* < 0.05, ** *p* < 0.01, and *** *p* < 0.001 indicate significance compared with the LPS group.

An MTT assay was performed to evaluate the impact of different concentrations of ombuin (the chemical structure shown in Figure 1A) on the viability of BV-2 cells, both with and without LPS stimulation. The results demonstrated that ombuin at concentrations ranging from 10 to 50 μM did not exhibit any cytotoxic effects on BV-2 cells, regardless of the presence or absence of LPS (Figure 1B,C). However, a dose of 100 μM of ombuin exhibited a detrimental effect, significantly reducing the cell viability of BV-2 cells in the presence of LPS (Figure 1C). Therefore, concentrations of 10 μM, 30 μM, and 50 μM of ombuin were selected for subsequent dose-dependent experiments. The anti-inflammatory effect of ombuin on microglial activation was then investigated. Upon LPS stimulation, pretreatment with ombuin significantly reduced the production of pro-inflammatory mediators NO (Figure 1D) and ROS (Figure 1H,I), as well as the release levels of cytokines IL-6 (Figure 1E), IL-1β (Figure 1F), and TNF-α (Figure 1G) in a dose-dependent manner.

### 2.2. Network Pharmacology Predicted the Targets for Neuroinflammation of Ombuin

**Figure 2:**
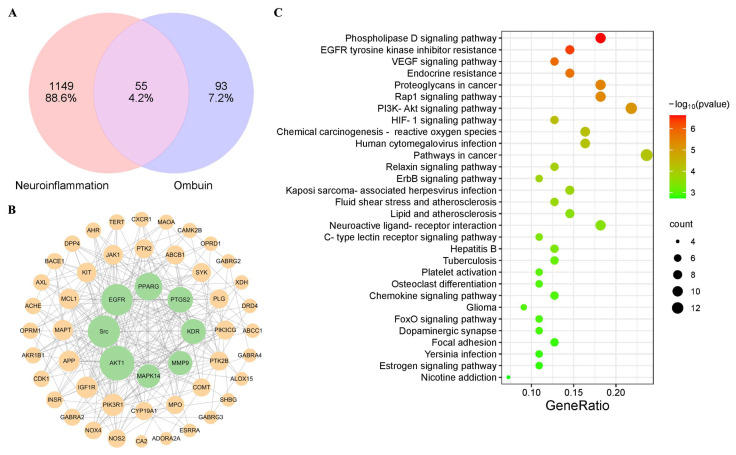
Network pharmacology was utilized to predict the targets of ombuin in neuroinflammation. (**A**) Neuroinflammation potential therapeutic targets vs. ombuin targets. (**B**) The protein–protein interaction (PPI) network of the potential therapeutic targets for neuroinflammation affected by ombuin. (**C**) KEGG Enrichment Scatter Plot displaying the top 30 KEGG pathways, with pathways listed in a bubble chart format.

A total of 148 potential targets of ombuin were predicted using databases such as ETCM, SymMap, and Swiss Target Prediction. Among these, 55 therapeutic targets were identified by intersecting with neuroinflammatory targets (Figure 2A). Subsequently, the interactions between these targets were analyzed by constructing a protein–protein interaction (PPI) network, which consisted of 52 nodes and 269 edges (Figure 2B). The major targets identified in the network include Src, AKT1, EGFR, PPARG, etc. The KEGG enrichment analysis identified a total of 87 signaling pathways. The top 30 enriched pathways revealed that ombuin potentially modulates neuroinflammation through various signaling cascades, including VEGF, Rap1, and PI3K-AKT pathways (Figure 2C).

### 2.3. Therapeutic Network of Ombuin in Neuroinflammatory Diseases

**Figure 3:**
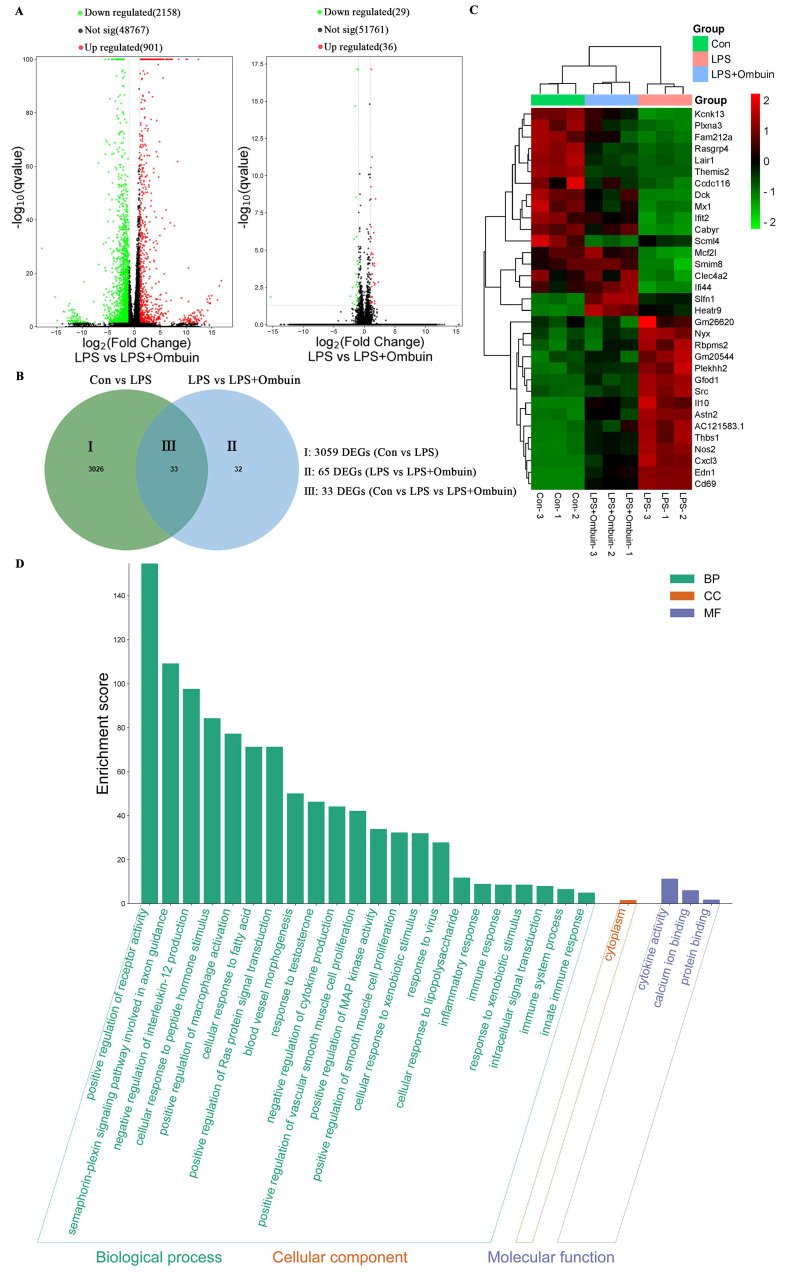
Analysis of Differentially Expressed Genes (DEGs) by Transcriptomics. (**A**,**B**) Volcano plot analysis of all DEGs among the control group, the LPS group, and the LPS + ombuin (50 μM) group. *n* = 3. (**C**) The heat map displays the 33 overlapping genes. In this diagram, red indicates higher expression levels, and green indicates lower expression levels. (**D**) Gene ontology (GO) enrichment analysis.

To elucidate the key targets of ombuin in mitigating LPS-induced neuroinflammation, RNA sequencing analysis was conducted, and a total of 33 differentially expressed genes (DEGs) were identified. Among these DEGs, 30 genes exhibited contrasting expression patterns upon the addition of ombuin following LPS modeling (Figure 3A,B). These genes are considered potential therapeutic targets for the treatment of neuroinflammation. The heat map depicted distinct colors, representing the overlapping set of these 33 genes across the three groups (Figure 3C). Furthermore, gene ontology (GO) enrichment analysis was performed to assess the biological functions encompassing biological processes, cellular components, and molecular functions associated with these DEGs. The results showed significant enrichment of the DEGs in categories such as receptor activity regulation, semaphorin–plexin signaling pathway, macrophage activation, and interleukin production (Figure 3D).

**Figure 4:**
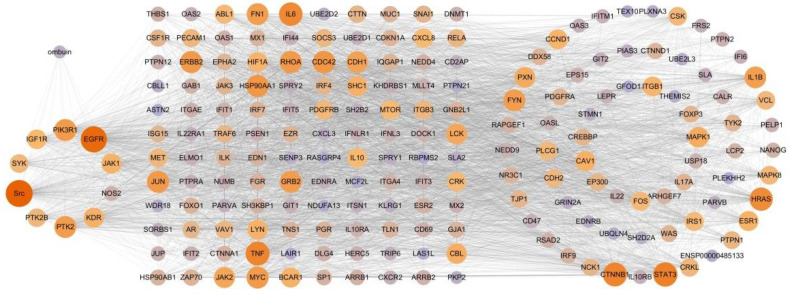
Network of ombuin and 199 targets.

The transcriptomics results reflected regulatory states in a specific context rather than the entire therapeutic network [27]. The String database, along with an extension of the PPI network, was utilized to identify the entire therapeutic network of ombuin in treating LPS-induced neuroinflammation. This expansion resulted in 206 potential therapeutic targets, including 26 additional targets (GM26620, AC121583.1, GM20544, and CLEC4A2 genes not recorded in String), which further supports the construction of the therapeutic networks for ombuin in neuroinflammation treatment. By intersecting the common targets between ombuin and therapeutic targets of neuroinflammation, we identified 10 core targets. Finally, a “component-core targets-potential therapeutic targets” network was constructed to gain a deeper understanding of the relationship between ombuin and the therapeutic network. This comprehensive therapeutic network consists of 200 nodes (ombuin, 10 core targets, and 189 potential therapeutic targets) connected by 4059 edges (Figure 4).

### 2.4. Potential Mechanism of Anti-Neuroinflammation of Ombuin

**Figure 5:**
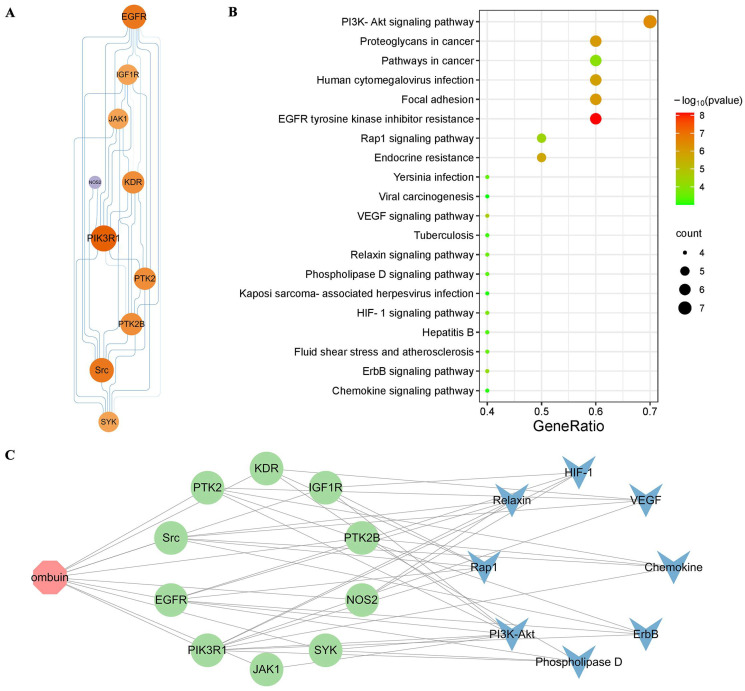
(**A**) PPI network of core targets. (**B**) KEGG analysis, the size and color of a node are indicative of its degree in the network; nodes with a larger size and a deeper orange color represent targets with higher degrees. (**C**) The ombuin-targets-pathways network illustrates ombuin’s role in the treatment of neuroinflammation.

The PPI network and detailed information on 10 core targets are shown in Figure 5A, illustrating the topological organization. The top five nodes, ranked by degree, namely PIK3R1, EGFR, Src, PTK2, and KDR, are considered hub targets in the treatment of neuroinflammation with ombuin. The top 20 pathways were visualized based on KEGG enrichment analysis (Figure 5B). Among them, the PI3K-AKT signaling pathway exhibited significant enrichment, suggesting its crucial role in neuroinflammation treatment. Figure 5C illustrates the component–target–pathway network of the ombuin for treating neuroinflammation, comprising 19 nodes (1 component, 10 core targets, and 8 signaling pathways) connected by a total of 46 edges. These findings indicate that ombuin might exert its effects on LPS-induced neuroinflammation treatment through multiple targets and pathways.

**Figure 6:**
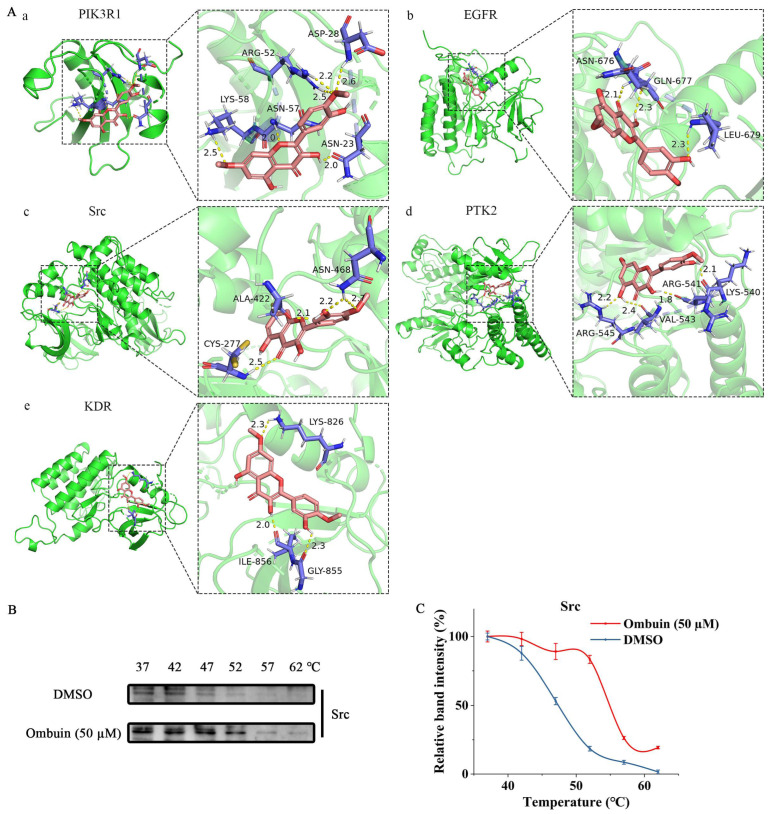
Src is a direct target of ombuin. (**A**) Molecular docking patterns of key targets with ombuin: (**a**) PIK3R1, (**b**) EGFR, (**c**) Src, (**d**) PTK2, (**e**) KDR. (**B**) BV-2 cells were pre-treated with 50 μM ombuin for 4 h, after which CETSA was performed using a temperature gradient ranging from 37 °C to 62 °C. The lysates were analyzed by Western blot with an Src antibody. (**C**) Relative band intensity. The expression levels of the Src protein in each group at different temperatures were normalized by dividing by the gray scan value of the Src protein at 37 °C to calculate the relative band intensity.

The molecular docking simulations were performed for the top 5 core targets with the highest degree values to verify the interaction between ombuin and these targets. The binding energies of all ingredient–target interactions, including specific binding sites, distances, and atoms, were below 0 kJ/mol, indicating a spontaneous binding ability between these core ingredients and targets. Importantly, all five targets in our experiment demonstrated negative binding energies with ombuin, suggesting spontaneous binding. Figure 6A illustrates the molecular docking patterns. Ombuin formed six hydrogen bonds with PIK3R1 (Figure 6A(a)), including a strong bond at 2.6 Å with ASP-28, bonds at 2.2 Å and 2.5 Å with ARG-52, a strong bond at 2.0 Å with both ASN-23 and ASN-57, and a strong bond at 2.5 Å with LYS-58. The binding energy of ombuin to PI3KR1 was −5.28 kcal/mol. Ombuin interacts with EGFR (Figure 6A(b)) through three hydrogen bonds with ASN-676, GLN-677 and LEU-679. The lengths of the hydrogen bonds were 2.1 Å, 2.3 Å, and 2.3 Å, respectively, and the binding energy was −4.34 kcal/mol. Ombuin and Src formed the most stable conformation with a binding energy of −5.96 kcal/mol (Figure 6A(c)), while the binding energies between ombuin and PTK2 and KDR were −4.66 and −4.24 kcal/mol, respectively (Figure 6A(d,e)). Furthermore, cell heat transfer analysis (CETSA) demonstrated that, compared to the control group, ombuin increased the thermal stability of the Src protein and prevented its temperature-dependent degradation (Figure 6B,C). These findings provide evidence supporting the role of Src as a direct target of ombuin, potentially contributing to its anti-neuroinflammatory effects in BV-2 cells.

### 2.5. Src Was Necessary for Ombuin-Mediated Anti-Inflammatory Activity

**Figure 7:**
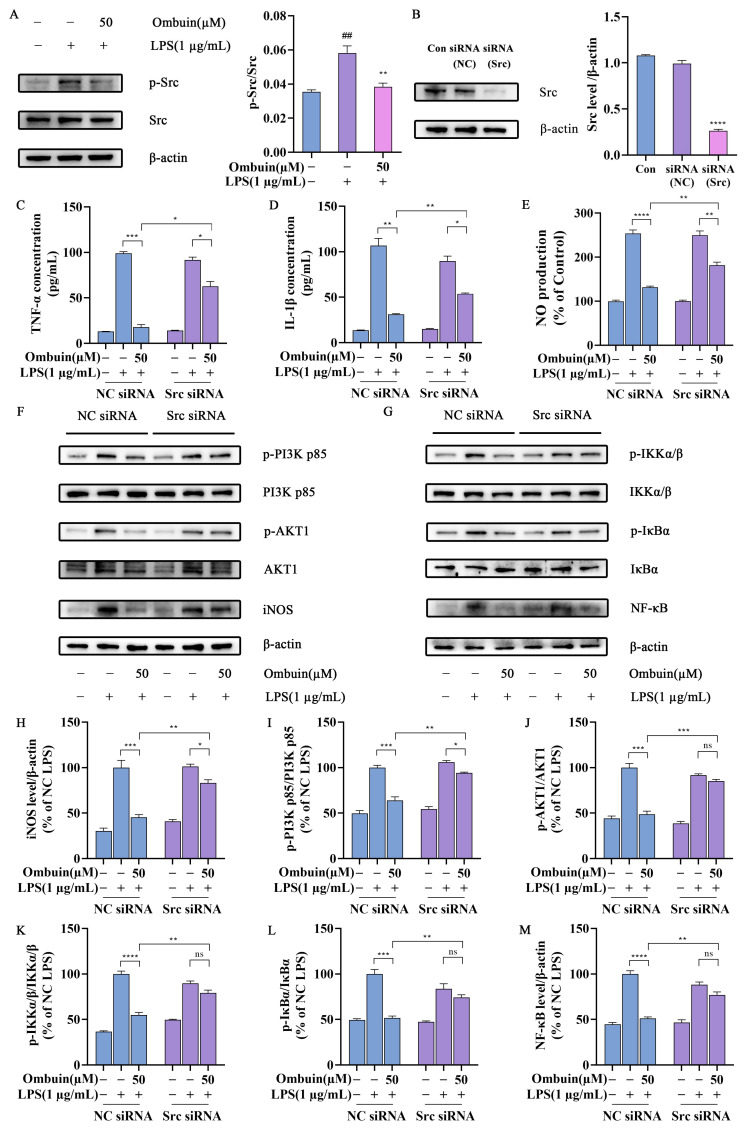
Src knockdown reversed ombuin-mediated neuroinflammatory inhibition. (**A**) The levels of Src and phosphorylated Src (p-Src) proteins were assessed using Western blot analysis following a 24 h treatment with ombuin and/or LPS. (**B**) The efficiency of Src siRNA transfection in BV-2 cells. (**C**–**E**) BV-2 cells were transfected with either non-targeting control (NC) siRNA or Src siRNA. After 24 h, the cells were further treated with ombuin/LPS for an additional 24 h. Then, the impact of Src knockdown on the production of TNF-α (**C**), IL-1β (**D**), and NO (**E**) in BV-2 cells was measured by ELISA. (**F**,**G**) Western blot analysis was used to evaluate the protein levels of the PI3K-AKT pathway, the NF-κB signaling pathway, and inducible nitric oxide synthase (iNOS) in BV-2 cells post-Src knockdown. (**H**–**M**) Quantitative analysis of the influence of Src silencing on downstream PI3K-AKT and NF-κB signaling pathways, as well as the expression of the inflammatory mediator iNOS protein. Data are presented as the mean ± SEM of three independent experiments. ## *p* < 0.01 indicates significance compared with the control group; * *p* < 0.05, ** *p* < 0.01, *** *p* < 0.001, and **** *p* < 0.0001 indicate significance; ‘ns’ indicates not significant.

To further investigate the underlying mechanism by which ombuin exerts its anti-neuroinflammatory effects through interaction with Src, immunoblotting analysis was performed to evaluate the impact of ombuin on Src protein. Our results revealed that LPS significantly enhanced Src phosphorylation compared to the control group; however, treatment with ombuin markedly suppressed Src phosphorylation (Figure 7A). These findings suggest that ombuin has the ability to inhibit Src function by blocking its phosphorylation. Subsequently, Src siRNA was transfected into BV-2 cells, and a significant reduction in Src protein expression was observed (Figure 7B). Upon LPS stimulation, we examined NO expression in BV-2 cells treated with or without ombuin and found that silencing of Src effectively reversed the inhibitory effect of ombuin on NO production (Figure 7C). Furthermore, a similar reversal effect of ombuin on TNF-α and IL-1β release was also observed in BV-2 cells transfected with Src siRNA (Figure 7D,E). The increased protein expression levels of p-PI3K p85, p-AKT1, p-IKKα/β, p-IκBα, NF-κB p65, and inducible nitric oxide synthase (iNOS) induced by LPS were inhibited by ombuin; however, this inhibition was reversed when Src was knocked down (Figure 7F–M). These results indicate that Src is the primary target through which ombuin exerts its anti-inflammatory effects in LPS-induced BV-2 cells.

## 3. Discussion

Neuroinflammation is an inflammatory reaction within the central nervous system, characterized by excessive microglial activation, and it plays a pivotal role in the pathogenesis of various diseases [28,29,30,31]. Natural products are known for their anti-inflammatory activities, often with fewer or no side effects compared to traditional synthetic drugs. These products have yielded numerous promising lead compounds with significant potential, such as aspirin and artemisinin, which have unique pharmacological mechanisms [32,33,34,35]. Investigating the mechanisms of bioactive natural products can aid in exploring novel therapeutic targets and provide valuable biological insights for drug development, particularly in the context of neurodegenerative diseases.

Various flavonoids have been reported to have the potential to counteract neurotoxicity and delay the onset of neuroinflammation via multiple targets and pathways [36,37]. Our findings demonstrated that ombuin, a main flavonoid compound isolated from *R. erythroxylon* Pall. leaves, exhibited potent anti-neuroinflammatory effects. Activated microglia induce the expression of inducible nitric oxide synthase (iNOS), catalyzing the production of substantial amounts of NO from L-arginine [38]. NO, a crucial inflammatory mediator, disrupts body homeostasis when imbalanced, contributing to the pathogenesis of various inflammatory diseases [39,40,41]. A previous study demonstrated that ombuin attenuated NO production in macrophages activated by LPS and interferon-gamma (IFN-γ) [26]. Our study observed that ombuin inhibited LPS-induced NO production in BV-2 cells in a concentration-dependent manner, offering preliminary evidence of its anti-neuroinflammatory potential. Pro-inflammatory factors such as TNF-α, IL-6, and IL-1β, released by activated microglia during neuroinflammation, are pivotal in initiating inflammatory responses, regulating cytokine cascades, and being implicated in neurodegenerative diseases [42,43,44]. In our study, ombuin demonstrated inhibitory effects on LPS-induced production of TNF-α, IL-6, and IL-1β in BV-2 cells. Additionally, we investigated ROS expression, an upstream regulator of cytokine-related signaling pathways that contribute to neurotoxicity and are induced by excessive inflammation [45,46]. Using DCFH-DA as a fluorescent probe, we determined that ombuin inhibited ROS production in LPS-treated BV-2 cells in a concentration-dependent manner. Ombuin’s potential anti-neuroinflammatory properties suggest it may be a promising candidate for treating neuroinflammation.

Next, we employed network pharmacology in conjunction with transcriptomics to identify potential targets of ombuin. Network pharmacology, a novel approach for elucidating the mechanisms of action of compounds, facilitates the prediction of compound-target interactions and sheds light on new drug development pathways [47]. However, it is important to note that targets identified through network pharmacology are primarily sourced from databases and may be subject to inherent biases. To provide a more accurate understanding of ombuin’s role in treating neuroinflammation, this study combined network pharmacology with transcriptomics [48]. By utilizing existing databases, we identified 55 targets related to ombuin for neuroinflammation treatment. Additionally, therapeutic targets were determined through transcriptomics analysis of LPS-induced BV-2 cells. To achieve a comprehensive view of ombuin’s regulatory network in neuroinflammation treatment, we expanded the protein–protein interaction (PPI) network using the String database, identifying 206 potential therapeutic targets. Subsequently, the intersection of the common and potential therapeutic targets associated with ombuin in neuroinflammation treatment yielded 10 key targets. These targets were considered promising candidates for ombuin. Among these, PIK3R1, EGFR, Src, PTK2, and KDR emerged as the top five proteins. Previous studies have shown that these proteins are significantly associated with neuroinflammation. PIK3R1, encoding the regulatory subunit of PI3K, suppresses PI3Kα activity in the absence of receptor tyrosine kinase stimulation and is crucial for neuronal function and neurotoxicity [49]. Epidermal growth factor receptor (EGFR) signaling is essential for microglial activation and response to neuroinflammatory stimuli, modulating the downstream NF-κB signaling pathway. Inhibiting EGFR signaling can effectively reduce microglial activation and curb the production of inflammatory factors, thus alleviating secondary neuroinflammatory damage [50]. PTK2 is closely associated with neuroinflammatory regulation and significantly influences the progression of neurodegenerative diseases [51]. KDR, also known as vascular endothelial growth factor receptor 2 (VEGFR-2), is linked to various neurodegenerative diseases, including Alzheimer’s, and affects microglial function and neuroinflammation processes, particularly through the regulation of downstream signaling pathways like PI3K/Akt [52]. Src is a key mediator in TLR4-induced inflammatory pathways, integrating multiple signals triggered by LPS or other stimuli, and acts as an upstream regulator of both the PI3K-AKT and NF-κB pathways, modulating inflammation [53]. KEGG enrichment analysis on the key targets established a “component–target–pathway” network for neuroinflammation treatment. Our findings suggest that the PI3K-AKT signaling pathway may be significantly involved in the regulation of neuroinflammation by ombuin. The polarization of microglia in neuroinflammation is closely related to the PI3K-AKT signaling pathway [54]. Therefore, our findings indicate that these five proteins are likely closely related to the anti-neuroinflammatory effects of ombuin. To validate the target prediction results, molecular docking experiments were conducted with ombuin and the five major proteins. These experiments showed that ombuin and Src formed the most stable complex, and CETSA experiments further confirmed that ombuin could enhance the thermal stability of the Src protein, preventing its temperature-dependent degradation.

The Src tyrosine kinase is crucial for cellular signal transduction and particularly significant in inflammatory responses. Increased activity of Src kinase, notably its phosphorylation at the Tyr416 site, indicates heightened functionality. Studies have shown that disrupting Src can inhibit LPS-induced NF-κB translocation in macrophages and reduce the release of pro-inflammatory factors [55]. Additionally, suppressing Src kinase activity has been observed to reduce amyloid-associated microglia proliferation both in vivo and in vitro [56]. In this study, ombuin treatment significantly inhibited the upregulation of phosphorylated Src. This phosphorylation is key to activating Src kinase and its downstream signaling pathways, including the critical PI3K/AKT signaling pathway. The PI3K/AKT pathway, once activated, is essential for sustaining cell survival, promoting cell proliferation, and regulating cellular metabolism [57]. PI3K, upon activation, generates phosphatidylinositol-3,4,5-trisphosphate (PIP3), which in turn recruits and activates AKT [58]. Our research demonstrated ombuin’s effectiveness in inhibiting the LPS-induced activation of the PI3K-AKT signaling pathway. Notably, reducing Src gene expression significantly diminished ombuin’s ability to inhibit the expression of p-PI3K p85 and p-AKT proteins, leading to a significant reduction in its inhibitory impact on the PI3K-AKT signaling pathway.

The activation of AKT is closely associated with the NF-κB signaling pathway during inflammatory responses [11]. Additionally, ROS act as signaling molecules that affect various pathways, including the IKK complex activation through oxidative stress, thus facilitating NF-κB activation [59]. NF-κB, a key transcription factor in regulating inflammatory responses, is bound to IκB proteins and remains in the cytoplasm when inactive. Upon stimulation by inflammatory signals, the IKK complex, which includes IKKα and IKKβ, activates and phosphorylates IκBα. This results in the degradation of IκBα and the subsequent release of NF-κB [60]. The released NF-κB translocates to the nucleus, where it triggers the expression of pro-inflammatory factors such as TNF-α, IL-6, and IL-1β, amplifying the inflammatory response. Our results found that ombuin significantly reduced the expression levels of NF-κB, p-IKKα/β, and p-IκBα, as well as the release of pro-inflammatory factors TNF-α and IL-1β in BV-2 cells stimulated by LPS. Furthermore, suppressing Src activity markedly blocked the inhibitory effect of ombuin on LPS-induced NF-κB signaling pathway activation. The activation of iNOS and the resulting production of NO are integral to the inflammatory response. While iNOS activation and the consequent increase in NO levels are crucial for inflammation and immune regulation, excessive NO in an inflammatory context can cause cytotoxicity and tissue damage [61]. Our findings indicated that ombuin treatment effectively suppressed the LPS-induced upregulation of iNOS protein and NO release. Moreover, silencing Src significantly diminished ombuin’s inhibitory effect on these processes. This finding underscored the critical role of Src in mediating ombuin’s anti-neuroinflammatory effects.

**Figure 8:**
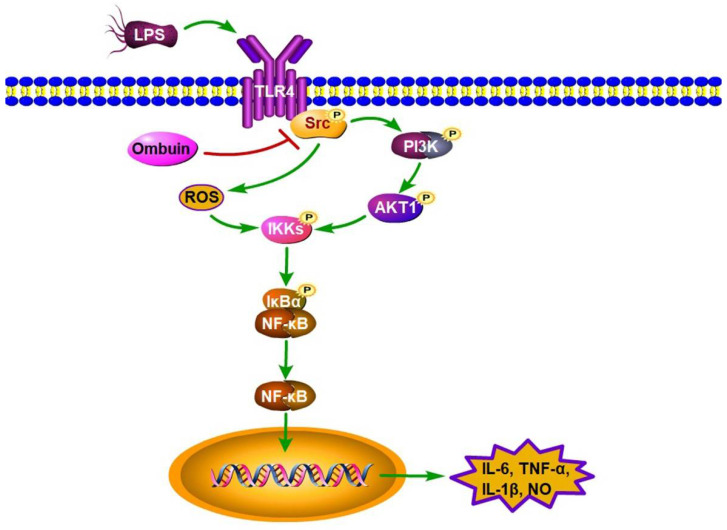
The proposed signaling mechanism explains the effects of ombuin on LPS-induced neuroinflammation in BV-2 cells.

In summary, our research revealed that ombuin directly binds to its target, Src, and effectively inhibits Src phosphorylation. This action subsequently blocked the activation of the LPS-induced PI3K/AKT signaling pathway in BV-2 cells. Furthermore, ombuin suppressed the NF-κB signaling pathway by regulating ROS production and reducing IKKα/β phosphorylation levels. As a result, it significantly attenuated the release of inflammatory factors and prevented LPS-induced neurotoxicity, demonstrating its potential as an anti-neuroinflammatory agent. These findings are summarized in Figure 8. Notably, ombuin exerts an anti-neuroinflammatory effect through a Src-mediated mechanism, offering a promising novel strategy for the treatment of neurodegenerative diseases related to inflammation.

## 4. Materials and Methods

### 4.1. Materials

The minimum essential medium (MEM) was purchased from Procell (Wuhan, China). Fetal bovine serum (FBS) was obtained from ExCell Bio (Suzhou, China). LPS (055: B5) was bought from Sigma-Aldrich (St. Louis, MO, USA). 3-(4,5-dimethylthiazol-2-yl)-2,5-diphenyltetrazolium bromide (MTT) was obtained from Solarbio (Beijing, China). The ROS Assay Kit was purchased from Beyotime (Shanghai, China). The Griess reagent was bought from ABclonal (Wuhan, China). Enzyme-linked immunosorbent assay (ELISA) kits for IL-1β, TNF-α, and IL-6 were procured from Elabscience (Shanghai, China).

### 4.2. Cell Culture

Mouse BV-2 cells, obtained from Procell (Wuhan, China), were cultured in MEM supplemented with 10% FBS and a penicillin–streptomycin mixture and then incubated in an environment with 5% CO_2_ at a temperature of 37 °C.

### 4.3. MTT Test

Ombuin was treated either alone or after pretreatment for 1 h and then co-cultured with LPS (1 μg/mL) for 24 h. Cell viability was assessed using the MTT reduction assay, following the protocol described previously [45]. MTT (5 mg/mL) solution was added to the cells and incubated for 4 h. Subsequently, DMSO (150 μL/well) was added to dissolve the purple crystals away from light. The optical density (OD) value at 570 nm was then measured using a microplate spectrophotometer (Thermo Fisher Scientific, Vantaa, Finland) [62].

### 4.4. Griess Test

NO content was determined using the Griess method, as detailed in a previous publication [63]. The cell supernatant was incubated with Griess reagent in a dark environment at 37 °C for 15 min, after which the absorbance at 450 nm was measured using a microplate reader. The NO concentration was calculated by substituting the measured absorbance values into a pre-established standard curve.

### 4.5. Measurement of Pro-Inflammatory Factors

Pro-inflammatory factor levels were determined using an ELISA kit, following the manufacturer’s protocol. The OD value was measured at 450 nm according to the instructions, and the sample concentration was calculated by reference to a standard curve.

### 4.6. ROS Detection Assay

ROS release was quantified using DCFH-DA, according to procedures described in previous studies [64]. BV-2 cells were treated with various concentrations of ombuin (10, 30, 50 μM) for 1 h, then incubated with or without LPS for 24 h. Next, the cells were incubated with 10 μM DCFH-DA for 20 min in darkness. After being washed with PBS, the cells’ green fluorescence was measured using laser confocal microscopy with 485 nm excitation and 535 nm emission. ImageJ software (version 1.52a, National Institutes of Health, Bethesda, MD, USA) analyzed the fluorescence intensity of the images. ROS levels were reported as multiples relative to the control group.

### 4.7. Network Pharmacology Analysis

To identify ombuin targets, databases including ETCM (https://www.tcmip.cn/ETCM/, accessed on 12 July 2023), SymMap (https://www.symmap.org/, accessed on 12 July 2023), and SwissTargetPrediction (https://swisstargetprediction.ch, accessed on 12 July 2023) were utilized for obtaining potential targets and eliminating duplicates. GeneCards (https://www.genecards.org/, accessed on 18 July 2023) and DisGeNET (https://www.disgenet.org/, accessed on 18 July 2023) were then integrated to identify targets related to neuroinflammation, creating a neuroinflammation-specific target library through a combination and deduplication process. The common targets between ombuin and neuroinflammation were identified by intersecting these libraries. Subsequently, an interaction network diagram was constructed using the String 11.5 database (https://string-db.org/, accessed on 25 July 2023) and Cytoscape 3.10.0 software. Pathway enrichment analysis was conducted using the Kyoto Encyclopedia of Genes and Genomes (KEGG), with a significance threshold of *p* < 0.05.

### 4.8. Transcriptome Analysis

The transcriptomic detection and analysis were conducted by Shanghai Sangon Biotech (Shanghai, China). Differentially expressed genes (DEGs) were screened using the criteria of |log_2_Fold Change| > 1 and *q*-Value < 0.05 to determine significance. Subsequently, an intersection analysis was carried out to obtain common DEGs. Gene ontology (GO) functional enrichment analysis was conducted with a significance threshold of *p* < 0.05. Additionally, a protein–protein interaction (PPI) network was constructed using the String database to further explore the potential therapeutic targets of ombuin.

### 4.9. Building a “Component–Target–Pathway” Network

The core targets for ombuin’s role in treating neuroinflammation were identified by intersecting the co-targets of ombuin and neuroinflammation with potential therapeutic targets. Cytoscape software was utilized to construct a network diagram that illustrates the relationships among “components”, “core targets”, and “potential targets”. This network was then analyzed topologically using the “Network Analysis” tool. Additionally, all core targets were analyzed using KEGG for enrichment to identify the signaling pathways associated with ombuin’s therapeutic effects on neuroinflammation. Based on this analysis, a network diagram depicting the “component–target–pathway” connections was constructed.

### 4.10. Molecular Docking

Docking simulations were conducted using Autodock software (version 4.2), and the results were visualized using PyMol software v2.6.0a0. Additionally, 3D structure files for the hub targets and ombuin were obtained from the Protein Data Bank (PDB) database (https://www.rcsb.org/, accessed on 4 October 2023).

### 4.11. Cell Heat Transfer Analysis (CETSA)

The cells were treated with 50 μM ombuin for 4 h. Subsequently, aliquots of the cells were heated at temperatures ranging from 37 to 62 °C for 3 min and then cooled to room temperature for another 3 min. For protein extraction, the cells were subjected to three cycles of freezing and thawing, alternating between liquid nitrogen and room temperature. Finally, centrifugation was performed at 20,000× *g* for 20 min, after which Western blot analysis was conducted.

### 4.12. Western Blot

BV2 cells were seeded in a 6-well plate. After treatment, the cells were washed with PBS two or three times, then lysed using RIPA Lysis buffer for 5 min with a protease inhibitor added a few minutes prior to use. Protein samples were diluted with protein loading buffer and boiled for 10 min at 100 °C. Equal amounts of protein (30 μg) were electrophoretically separated by SDS-PAGE and transferred onto nitrocellulose filter membranes (Biosharp, Hefei, China). Membranes were blocked with 5% BSA in TBST (20 mM Tris, 150 mM NaCl, and 0.1% Tween 20) for 1 h at room temperature, then incubated with specific primary antibodies in 2% BSA in TBST overnight at 4 °C. The primary antibodies used included NF-κB p65 (1:1000, ABclonal, Wuhan, China), iNOS (1:1000, ABclonal), Src (1:1000, ABclonal), p-Src (1:1000, ABclonal), PI3K p85 (1:1000, ABclonal), p-PI3K p85 (1:1000, ABclonal), AKT1 (1:1000, ABclonal), p-AKT1 (1:1000, ABclonal), IKKα/β (1:1000, HuaBio, Hangzhou, China), p-IKKα/β (1:1000, ABclonal), IκBα (1:1000, ABclonal), p-IκBα (1:1000, ABclonal), and β-actin (1:1000, ABclonal). After washing with TBST, the membranes were incubated with HRP-conjugated secondary antibodies (1:5000, ABclonal) in 2% BSA in TBST for 1 h at room temperature. The proteins transferred onto membranes were detected using the SuperKine™ West Femto Maximum Sensitivity Substrate (Abbkine, Wuhan, China). Visualization was performed using the Bio-Rad ChemiDoc MP Imaging system (Bio-Rad, Hercules, CA, USA), and the protein band intensity was quantified using Image Lab software v6.0.0.

### 4.13. Src siRNA Transfection

The expression of Src in cells was inhibited using the TransIntro^TM^ EL transfection reagent (TransGen Biotech, Beijing, China). Both Src-siRNA (sense: GCCUAAAUGUGAAACACUATT, anti-sense: UAGUGUUUCACAUUUAGGCTT) and scrambled siRNA (sense: UUCUCCGAACGUGUCACGUTT, anti-sense: ACGUGACACGUUCGGAGAATT) were constructed by GenePharma (Shanghai, China). The siRNAs were transfected into BV-2 cells according to the manufacturer’s instructions, and the efficiency of transfection was evaluated by Western Blot analysis.

### 4.14. Statistical Analysis

The statistical analysis and graphing were conducted using Graph Pad Prism 8.0 software (San Diego, CA, USA) and Origin 8 (Nampton, MA, USA). The Shapiro–Wilk test was used to assess the normality of the data before applying parametric tests. The data were presented as mean ± standard error (mean ± SEM), and results were analyzed by one-way analysis of variance (ANOVA) followed by Bonferroni’s multiple comparisons test. A value of *p* < 0.05 was considered statistically significant.

## 5. Conclusions

Generally speaking, this study demonstrates that ombuin can exert an anti-neuroinflammatory effect, and the mechanism may be related to the inhibition of its direct target Src phosphorylation and the suppression of the downstream PI3K-AKT/NF-κB pathways. The findings elucidate the molecular mechanisms underlying ombuin’s anti-neuroinflammatory effects, providing valuable insights for pharmacological interventions aimed at neuroinflammation and aiding in the development of novel natural active compounds. Additionally, these findings also provide a new reference point for further utilization of *R. erythroxylon* Pall. in the formulation of functional beverages and pharmaceutical products.

## Data Availability

Data will be made available upon request.

## References

[1] Woodburn S.C., Bollinger J.L., Wohleb E.S. (2021). The semantics of microglia activation: Neuroinflammation, homeostasis, and stress. J. Neuroinflamm..

[2] Maher P. (2019). The Potential of Flavonoids for the Treatment of Neurodegenerative Diseases. Int. J. Mol. Sci..

[3] Li S.Y., Zhou Y.L., He D.H., Liu W., Fan X.Z., Wang Q., Pan H.F., Cheng Y.X., Liu Y.Q. (2020). Centipeda minima extract exerts antineuroinflammatory effects via the inhibition of NF-κB signaling pathway. Phytomedicine.

[4] Singh D. (2022). Astrocytic and microglial cells as the modulators of neuroinflammation in Alzheimer’s disease. J. Neuroinflamm..

[5] Decandia D., Gelfo F., Landolfo E., Balsamo F., Petrosini L., Cutuli D. (2023). Dietary Protection against Cognitive Impairment, Neuroinflammation and Oxidative Stress in Alzheimer’s Disease Animal Models of Lipopolysaccharide-Induced Inflammation. Int. J. Mol. Sci..

[6] Stöberl N., Maguire E., Salis E., Shaw B., Hall-Roberts H. (2023). Human iPSC-derived glia models for the study of neuroinflammation. J. Neuroinflamm..

[7] Kwon H.S., Koh S.H. (2020). Neuroinflammation in neurodegenerative disorders: The roles of microglia and astrocytes. Transl. Neurodegener..

[8] Muzio L., Viotti A., Martino G. (2021). Microglia in neuroinflammation and neurodegeneration: From understanding to therapy. Front. Neurosci..

[9] Yu C.I., Cheng C.I., Kang Y.F., Chang P.C., Lin I.P., Kuo Y.H., Jhou A.J., Lin M.Y., Chen C.Y., Lee C.H. (2020). Hispidulin Inhibits Neuroinflammation in Lipopolysaccharide-Activated BV2 Microglia and Attenuates the Activation of Akt, NF-κB, and STAT3 Pathway. Neurotox. Res..

[10] Ismail E.N., Jantan I., Vidyadaran S., Jamal J.A., Azmi N. (2020). *Phyllanthus amarus* prevents LPS-mediated BV2 microglial activation via MyD88 and NF-κB signaling pathways. BMC Complement. Med. Ther..

[11] Jana M., Jana A., Liu X., Ghosh S., Pahan K. (2007). Involvement of phosphatidylinositol 3-kinase-mediated up-regulation of I kappa B alpha in anti-inflammatory effect of gemfibrozil in microglia. J. Immunol..

[12] Gandhi G.R., Mohana T., Athesh K., Hillary V.E., Vasconcelos A.B.S., de Franca M.N.F., Montalvão M.M., Ceasar S.A., Jothi G., Sridharan G. (2023). Anti-inflammatory natural products modulate interleukins and their related signaling markers in inflammatory bowel disease: A systematic review. J. Pharm. Anal..

[13] Xu G., Dong F., Su L., Tan Z.X., Lei M., Li L., Wen D., Zhang F. (2024). The role and therapeutic potential of nuclear factor κB (NF-κB) in ischemic stroke. Biomed. Pharmacother..

[14] Shin E.J., Hwang Y.G., Sharma N., Tran H.Q., Dang D.K., Jang C.G., Jeong J.H., Nah S.Y., Nabeshima T., Kim H.C. (2018). Role of protein kinase Cδ in dopaminergic neurotoxic events. Food Chem. Toxicol..

[15] Xu P., Huang M.W., Xiao C.X., Long F., Wang Y., Liu S.Y., Jia W.W., Wu W.J., Yang D., Hu J.F. (2017). Matairesinol Suppresses Neuroinflammation and Migration Associated with Src and ERK1/2-NF-κB Pathway in Activating BV2 Microglia. Neurochem. Res..

[16] Yang C.C., Lin C.C., Hsiao L.D., Kuo J.M., Tseng H.C., Yang C.M. (2020). Lipopolysaccharide-Induced Matrix Metalloproteinase-9 Expression Associated with Cell Migration in Rat Brain Astrocytes. Int. J. Mol. Sci..

[17] Lowell C.A. (2004). Src-family kinases: Rheostats of immune cell signaling. Mol. Immunol..

[18] Gage M., Putra M., Wachter L., Dishman K., Gard M., Gomez-Estrada C., Thippeswamy T. (2022). Saracatinib, a Src Tyrosine Kinase Inhibitor, as a Disease Modifier in the Rat DFP Model: Sex Differences, Neurobehavior, Gliosis, Neurodegeneration, and Nitro-Oxidative Stress. Antioxidants.

[19] Tai W., Ye X., Bao X., Zhao B., Wang X., Zhang D. (2013). Inhibition of Src tyrosine kinase activity by squamosamide derivative FLZ attenuates neuroinflammation in both in vivo and in vitro Parkinson’s disease models. Neuropharmacology.

[20] Bosco F., Ruga S., Citraro R., Leo A., Guarnieri L., Maiuolo J., Oppedisano F., Macrì R., Scarano F., Nucera S. (2023). The Effects of *Andrographis paniculata* (Burm.F.) Wall. Ex Nees and Andrographolide on Neuroinflammation in the Treatment of Neurodegenerative Diseases. Nutrients.

[21] Barber K., Mendonca P., Evans J.A., Soliman K.F.A. (2023). Antioxidant and Anti-Inflammatory Mechanisms of Cardamonin through Nrf2 Activation and NF-kB Suppression in LPS-Activated BV-2 Microglial Cells. Int. J. Mol. Sci..

[22] Elbandy M. (2023). Anti-Inflammatory Effects of Marine Bioactive Compounds and Their Potential as Functional Food Ingredients in the Prevention and Treatment of Neuroinflammatory Disorders. Molecules.

[23] Al-Azzawie H.F., Alhamdani M.S.S. (2006). Hypoglycemic and antioxidant effect of oleuropein in alloxan-diabetic rabbits. Life Sci..

[24] Nguyen T.T., Ketha A., Hieu H.V., Tatipamula V.B. (2021). In vitro antimycobacterial studies of flavonols from *Bauhinia vahlii* Wight and Arn. 3 Biotech.

[25] Rao Y.K., Fang S.H., Tzeng Y.M. (2008). Antiinflammatory activities of flavonoids and a triterpene caffeate isolated from *Bauhinia variegata*. Phytother. Res..

[26] Ranjana, Nooreen Z., Bushra U., Jyotshna, Bawankule D.U., Shanker K., Ahmad A., Tandon S. (2019). Standardization and xanthine oxidase inhibitory potential of *Zanthoxylum armatum* fruits. J. Ethnopharmacol..

[27] Dong Z., Chen Y. (2013). Transcriptomics: Advances and approaches. Sci. China Life Sci..

[28] Streit W.J., Mrak R.E., Griffin W.S.T. (2004). Microglia and neuroinflammation: A pathological perspective. J. Neuroinflamm..

[29] El-Bakoush A., Olajide O.A. (2018). Formononetin inhibits neuroinflammation and increases estrogen receptor beta (ERβ) protein expression in BV2 microglia. Int. Immunopharmacol..

[30] Zheng Y., Fang W., Fan S., Liao W., Xiong Y., Liao S., Li Y., Xiao S., Liu J. (2018). Neurotropin inhibits neuroinflammation via suppressing NF-κB and MAPKs signaling pathways in lipopolysaccharide-stimulated BV2 cells. J. Pharmacol. Sci..

[31] Badoer E. (2010). Microglia: Activation in acute and chronic inflammatory states and in response to cardiovascular dysfunction. Int. J. Biochem. Cell Biol..

[32] Karimi A., Majlesi M., Rafieian-Kopaei M. (2015). Herbal versus synthetic drugs; beliefs and facts. J. Nephropharmacol..

[33] So Y.J., Lee J.U., Yang G.S., Yang G., Kim S.W., Lee J.H., Kim J.U. (2024). The Potentiality of Natural Products and Herbal Medicine as Novel Medications for Parkinson’s Disease: A Promising Therapeutic Approach. Int. J. Mol. Sci..

[34] Zhang W., Xu H., Li C., Han B., Zhang Y. (2024). Exploring Chinese herbal medicine for ischemic stroke: Insights into microglia and signaling pathways. Front. Pharmacol..

[35] Ross F.C., Mayer D.E., Horn J., Cryan J.F., Del Rio D., Randolph E., Gill C.I.R., Gupta A., Ross R.P., Stanton C. (2024). Potential of dietary polyphenols for protection from age-related decline and neurodegeneration: A role for gut microbiota?. Nutr. Neurosci..

[36] He Y.Q., Zhou C.C., Jiang S.G., Lan W.Q., Zhang F., Tao X., Chen W.S. (2024). Natural products for the treatment of chemotherapy-related cognitive impairment and prospects of nose-to-brain drug delivery. Front. Pharmacol..

[37] Dias M.C., Pinto D.C.G.A., Silva A.M.S. (2021). Plant Flavonoids: Chemical Characteristics and Biological Activity. Molecules.

[38] Cianciulli A., Porro C., Calvello R., Trotta T., Lofrumento D.D., Panaro M.A. (2020). Microglia Mediated Neuroinflammation: Focus on PI3K Modulation. Biomolecules.

[39] Panneerselvan P., Vasanthakumar K., Muthuswamy K., Krishnan V., Subramaniam S. (2023). Insights on the functional dualism of nitric oxide in the hallmarks of cancer. Biochim. Biophys. Acta Rev. Cancer.

[40] Russell T.M., Richardson D.R. (2023). The good Samaritan glutathione-S-transferase P1: An evolving relationship in nitric oxide metabolism mediated by the direct interactions between multiple effector molecules. Redox Biol..

[41] Dilshara M.G., Lee K.T., Jayasooriya R.G.P.T., Kang C.H., Park S.R., Choi Y.H., Choi I.W., Hyun J.W., Chang W.Y., Kim Y.S. (2014). Downregulation of NO and PGE2 in LPS-stimulated BV2 microglial cells by trans-isoferulic acid via suppression of PI3K/Akt-dependent NF-κB and activation of Nrf2-mediated HO-1. Int. Immunopharmacol..

[42] Du R.W., Du R.H., Bu W.G. (2014). β-Arrestin 2 mediates the anti-inflammatory effects of fluoxetine in lipopolysaccharide-stimulated microglial cells. J. Neuroimmune Pharmacol..

[43] Ramsey C.P., Tansey M.G. (2014). A survey from 2012 of evidence for the role of neuroinflammation in neurotoxin animal models of Parkinson’s disease and potential molecular targets. Exp. Neurol..

[44] Wang X., Wang C., Wang J., Zhao S., Zhang K., Wang J., Zhang W., Wu C., Yang J. (2014). Pseudoginsenoside-F11 (PF11) exerts anti-neuroinflammatory effects on LPS-activated microglial cells by inhibiting TLR4-mediated TAK1/IKK/NF-κB, MAPKs and Akt signaling pathways. Neuropharmacology.

[45] Akanchise T., Angelova A. (2023). Potential of Nano-Antioxidants and Nanomedicine for Recovery from Neurological Disorders Linked to Long COVID Syndrome. Antioxidants.

[46] Patten D.A., Germain M., Kelly M.A., Slack R.S. (2010). Reactive oxygen species: Stuck in the middle of neurodegeneration. J. Alzheimers Dis..

[47] Hou Z., Sun L., Jiang Z., Zeng T., Wu P., Huang J., Liu H., Xiao P. (2024). Neuropharmacological insights into *Gardenia jasminoides* Ellis: Harnessing therapeutic potential for central nervous system disorders. Phytomedicine.

[48] Li A.P., He S.S., Zhang W.N., Zhang L.C., Liu Y.T., Li K., Qin X.M. (2020). Exploration the active compounds of Astragali Radix in treatment of adriamycin nephropathy by network pharmacology combined with transcriptomic approach. J. Ethnopharmacol..

[49] Sun Q., Wang H., Yang M., Xia H., Wu Y., Liu Q., Tang H. (2023). miR-153-3p via PIK3R1 Is Involved in Cigarette Smoke-Induced Neurotoxicity in the Brain. Toxics.

[50] Qu W.S., Tian D.S., Guo Z.B., Fang J., Zhang Q., Yu Z.Y., Xie M.J., Zhang H.Q., Lü J.G., Wang W. (2012). Inhibition of EGFR/MAPK signaling reduces microglial inflammatory response and the associated secondary damage in rats after spinal cord injury. J. Neuroinflamm..

[51] Lee S., Jo M., Kwon Y., Jeon Y.M., Kim S., Lee K.J., Kim H.J. (2023). PTK2 regulates tau-induced neurotoxicity via phosphorylation of p62 at Ser403. J. Neurogenet..

[52] Cohen J., Mathew A., Dourvetakis K.D., Sanchez-Guerrero E., Pangeni R.P., Gurusamy N., Aenlle K.K., Ravindran G., Twahir A., Isler D. (2024). Recent Research Trends in Neuroinflammatory and Neurodegenerative Disorders. Cells.

[53] Check J., Byrd C.L., Menio J., Rippe R.A., Hines I.N., Wheeler M.D. (2010). Src kinase participates in LPS-induced activation of NADPH oxidase. Mol. Immunol..

[54] Dong H., Zhang X., Dai X., Lu S., Gui B., Jin W., Zhang S., Zhang S., Qian Y. (2014). Lithium ameliorates lipopolysaccharide-induced microglial activation via inhibition of toll-like receptor 4 expression by activating the PI3K/Akt/FoxO1 pathway. J. Neuroinflamm..

[55] Khadaroo R.G., Kapus A., Powers K.A., Cybulsky M.I., Marshall J.C., Rotstein O.D. (2003). Oxidative stress reprograms lipopolysaccharide signaling via Src kinase-dependent pathway in RAW 264.7 macrophage cell line. J. Biol. Chem..

[56] Dhawan G., Combs C.K. (2012). Inhibition of Src kinase activity attenuates amyloid associated microgliosis in a murine model of Alzheimer’s disease. J. Neuroinflamm..

[57] Moghbeli M. (2024). PI3K/AKT pathway as a pivotal regulator of epithelial-mesenchymal transition in lung tumor cells. Cancer Cell Int..

[58] He Y., Sun M.M., Zhang G.G., Yang J., Chen K.S., Xu W.W., Li B. (2021). Targeting PI3K/Akt signal transduction for cancer therapy. Signal Transduct. Target. Ther..

[59] Guo Q., Jin Y., Chen X., Ye X., Shen X., Lin M., Zeng C., Zhou T., Zhang J. (2024). NF-κB in biology and targeted therapy: New insights and translational implications. Signal Transduct. Target. Ther..

[60] Li C., Liu M., Deng L., Luo D., Ma R., Lu Q. (2023). Oxyberberine ameliorates TNBS-induced colitis in rats through suppressing inflammation and oxidative stress via Keap1/Nrf2/NF-κB signaling pathways. Phytomedicine.

[61] Palmieri E.M., Gonzalez-Cotto M., Baseler W.A., Davies L.C., Ghesquière B., Maio N., Rice C.M., Rouault T.A., Cassel T., Higashi R.M. (2020). Nitric oxide orchestrates metabolic rewiring in M1 macrophages by targeting aconitase 2 and pyruvate dehydrogenase. Nat. Commun..

[62] Seo S.Y., Joo S.H., Lee S.-O., Yoon G., Cho S.-S., Choi Y.H., Park J.W., Shim J.-H. (2024). Activation of p38 and JNK by ROS Contributes to Deoxybouvardin-Mediated Intrinsic Apoptosis in Oxaliplatin-Sensitive and -Resistant Colorectal Cancer Cells. Antioxidants.

[63] Guo C., Yang L., Wan C.X., Xia Y.Z., Zhang C., Chen M.H., Wang Z.D., Li Z.R., Li X.M., Geng Y.D. (2016). Anti-neuroinflammatory effect of Sophoraflavanone G from *Sophora alopecuroides* in LPS-activated BV2 microglia by MAPK, JAK/STAT and Nrf2/HO-1 signaling pathways. Phytomedicine.

[64] Peng J.C., Deng Y., Song H.X., Fang Y.Y., Gan C.L., Lin J.J., Luo J.J., Zheng X.W., Aschner M., Jiang Y.M. (2023). Protective Effects of Sodium Para-Aminosalicylic Acid on Lead and Cadmium Co-Exposure in SH-SY5Y Cells. Brain Sci..

